# An Update on African Trypanocide Pharmaceutics and Resistance

**DOI:** 10.3389/fvets.2022.828111

**Published:** 2022-03-07

**Authors:** Keneth Iceland Kasozi, Ewan Thomas MacLeod, Ibrahim Ntulume, Susan Christina Welburn

**Affiliations:** ^1^Infection Medicine, Deanery of Biomedical Sciences, College of Medicine and Veterinary Medicine, The University of Edinburgh, Edinburgh, United Kingdom; ^2^School of Medicine, Kabale University, Kabale, Uganda; ^3^School of Biosecurity Biotechnical and Laboratory Sciences, College of Medicine and Veterinary Medicine, Makerere University, Kampala, Uganda; ^4^Zhejiang University-University of Edinburgh Joint Institute, Zhejiang University, Hangzhou, China

**Keywords:** trypanocides, trypanosoma, trypanosomiasis, drug resistance, HAT, AAT

## Abstract

African trypanosomiasis is associated with *Trypanosoma evansi, T. vivax, T. congolense*, and *T. brucei* pathogens in African animal trypanosomiasis (AAT) while *T. b gambiense* and *T. b rhodesiense* are responsible for chronic and acute human African trypanosomiasis (HAT), respectively. Suramin sodium suppresses ATP generation during the glycolytic pathway and is ineffective against *T. vivax* and *T. congolense* infections. Resistance to suramin is associated with pathogen altered transport proteins. Melarsoprol binds irreversibly with pyruvate kinase protein sulfhydryl groups and neutralizes enzymes which interrupts the trypanosome ATP generation. Melarsoprol resistance is associated with the adenine-adenosine transporter, P2, due to point mutations within this transporter. Eflornithine is used in combination with nifurtimox. Resistance to eflornithine is caused by the deletion or mutation of TbAAT6 gene which encodes the transmembrane amino acid transporter that delivers eflornithine into the cell, thus loss of transporter protein results in eflornithine resistance. Nifurtimox alone is regarded as a poor trypanocide, however, it is effective in melarsoprol-resistant gHAT patients. Resistance is associated with loss of a single copy of the genes encoding for nitroreductase enzymes. Fexinidazole is recommended for first-stage and non-severe second-stage illnesses in gHAT and resistance is associated with trypanosome bacterial nitroreductases which reduce fexinidazole. In AAT, quinapyramine sulfate interferes with DNA synthesis and suppression of cytoplasmic ribosomal activity in the mitochondria. Quinapyramine sulfate resistance is due to variations in the potential of the parasite's mitochondrial membrane. Pentamidines create cross-links between two adenines at 4–5 pairs apart in adenine-thymine-rich portions of *Trypanosoma* DNA. It also suppresses type II topoisomerase in the mitochondria of *Trypanosoma* parasites. Pentamidine resistance is due to loss of mitochondria transport proteins P2 and HAPT1. Diamidines are most effective against *Trypanosome brucei* group and act *via* the P2/TbAT1 transporters. Diminazene aceturate resistance is due to mutations that alter the activity of P2, TeDR40 (*T. b. evansi*). Isometamidium chloride is primarily employed in the early stages of trypanosomiasis and resistance is associated with diminazene resistance. Phenanthridine (homidium bromide, also known as ethidium bromide) acts by a breakdown of the kinetoplast network and homidium resistance is comparable to isometamidium. In humans, the development of resistance and adverse side effects against monotherapies has led to the adoption of nifurtimox-eflornithine combination therapy. Current efforts to develop new prodrug combinations of nifurtimox and eflornithine and nitroimidazole fexinidazole as well as benzoxaborole SCYX-7158 (AN5568) for HAT are in progress while little comparable progress has been done for the development of novel therapies to address trypanocide resistance in AAT.

## Introduction

Trypanosomiasis is caused by the parasite of the genus *Trypanosoma* (*T*.) and the disease affects both humans and animals (1). In humans, the disease is generally categorized based on the protozoan species, i.e., Chagas disease (American trypanosomiasis) and sleeping sickness (human African trypanosomiasis). Both forms of the disease are prevalent in low-income countries of America, Africa, and Asia and it is of major concern since it affects wildlife (2) and livestock such as equines (horses and camels), and large and small ruminants (i.e., bovines, ovines, and caprines) (3). This affects livestock productivity especially among horses, donkeys, camels, sheep, goats, and buffalo due to its associated economic burden (4). *Trypanosoma evansi, T. vivax, T. congolense*, and *T. brucei* are the major protozoan species that cause animal trypanosomiasis notably African animal trypanosomiasis (AAT) (5).

In developing countries of Latin America, Africa, and South East Asia, over 31 species of *Trypanosoma* infected tsetse flies have been identified (6). This continues to put the lives of over 60 million humans and 160 million animals in peril following a wave of infection in the affected communities (7). In Africa (40/54 countries), 55 million animals in the tsetse belt are at risk of AAT with annual mortality rates in cattle of 55 million, 30 million in sheep, and 40 million in goats (3). Livestock losses reduce livestock productivity since they are used for farm traction (8), and also as sources of community livelihood (9). Furthermore, human African trypanosomiasis (HAT) is present in (36/54) African countries. Angola, Cameroon, Central African Republic, Chad, Congo, Guinea, Malawi, South Sudan, and Zambia reported approximately 10 to 100 new cases in 2019, while Côte d'Ivoire, Equatorial Guinea, Gabon, Uganda, United Republic of Tanzania, and Zimbabwe reported 1 to 10 new cases (10). Furthermore, in the last 10 years, isolated instances have been documented in Burkina Faso, Ghana, Kenya, and Nigeria. In countries such as Benin, Botswana, Burundi, Ethiopia, Gambia, Guinea Bissau, Liberia, Mali, Mozambique, Namibia, Niger, Rwanda, Senegal, Sierra Leone, Swaziland, and Togo, however, no new cases have been registered in over a decade (10, 11). In Ethiopia, 8.6% of cases are caused by *T. congolense* (82 cases) and a few cases (3) are attributed to *T. vivax* (7). However, Uganda is the only country globally where both *T. brucei rhodesiense* (rHAT) and *T. brucei gambiense* (gHAT) have been reported (12).

Strategic control measures may have been implemented in such countries to halt transmission; but, due to poor monitoring and diagnostic activities, as well as inaccessibility and unstable socioeconomic conditions in such areas, it is impossible to determine the true incidence of disease at a particular point in time. This is important due to the close interactions between animals and humans across the tsetse belt region for the promotion of socioeconomic transformation in the affected communities (6).

## The Vector

The tsetse insect is a well-known biological vector for trypanosomiasis transmission. Tsetse flies belong to the genus Glossina, which is divided into three subgroups: *Glossina* (*G. morsitans* group), Austenina (*G. fusca* group), and Nemorhina (*G. palpalis* group). The ability to become infected while feeding on a vertebrate host, as well as the ability to sustain the development of the infection and transmit trypanosomes to a new vertebrate host, indicates the vectorial potential of *Glossina* species. According to these criteria, the *G. palpalis* and *G. morsitans* groups are the only ones that include *T. b. gambiense* vector species and subspecies. *G. palpalis palpalis* is common in forested areas of west and central Africa while *G. palpalis gambiensis* is prevalent in the savannah belt. Both species of *G. palpalis* group are effective *T. b. gambiense* vectors. *G.m. morsitans* and *G.m. centralis* in East Africa (excluding Uganda and Kenya), *G. pallidipes* in eastern and southern Africa, and *G. swynnertoni* in Kenya and Tanzania are the most common vectors for *T. b. rhodesiense* and are all prevalent in the savannah region.

Flies frequently visit forested regions and thickets throughout the savannah in eastern Africa, and woods and vegetation near streams in western Africa (3). In this environment, they can become infected during a blood meal on wildlife (2), and subsequently introduce infections in domestic livestock. In addition, both tsetse fly sexes have the capacity to carry and transmit infection (13). The ability of trypanosomes to cross the placenta has led to maternal-fetal infections of HAT (3).

In animals, tsetse flies may mechanically spread trypanosomes by starting a blood meal on one infected host and ending it on another, as long as there is a short interval between the two meals for parasites to thrive in the insect mouthparts (3, 14).

## The *Trypanosoma* Pathogen

Trypanosomes are a group of protozoan eukaryotes and their genomes and modes of gene expression differ in several important aspects from those of other eukaryotic model organisms (15). The African trypanosome, *Trypanosoma brucei* is 35 megabases with an extremely high diversity in different isolates. The chromosomal telomeres possess TTAGGG repeats and many of the telomeres of the megabases and intermediate chromosomes are linked to expression sites for genes encoding variant surface glycoproteins (VSG) showing that *T. brucei* has recent origins, and ancestral gene lineages have been repeatedly co-opted to novel functions (16). Furthermore, the C-terminal domain of *T. brucei* VSG plays a crucial role in facilitating exchange and metabolism, and acts as a barrier against chemotherapeutical agents (17, 18).

The minichromosomes serve as repositories for VSG genes since some of their telomeres are linked to non-transcribed copies of VSG genes and these expand the VSG gene pool, allowing the parasite to avoid elimination by the host immune system, a situation complicated further by the great VSG diversity in *Trypanosoma brucei* (19, 20). The multiplicity in the copies of VSG genes provides new insights since a single VSG protein is expressed from approximately 15 expression sites (ESs), proximal to the telomeres of megabase or intermediate chromosomes (21). *T. brucei* selective switching on and off of VSG involves converting VSG cassettes into active ES by diluting cell division and turnover being relatively much slower (22), demonstrating parasitic adaptations during host response to infection in an effort to evade the hosts immune defenses. The sequence of ES proteins showed mosaics and broad recombination, a strategy that has influenced its current evolutionary function and structure (23). Comparisons in *T. b. gambiense* and *T. b. brucei* sequences identified 99.2% similarity in coding regions and the gene order was collinear while a comparison in the VSG from both parasites showed that the structural repertoire of VSG domains was well-conserved across the two subspecies (24).

The “housekeeping” portion of the *T. brucei* genome is encompassed by 11 pairs of megabase-size chromosomes (MBCs) per genome (15, 21). Tolemere-associated chromosome fragmentation has shown that the GC-rich transcriptional “strand-swtich” is composed mainly of retrotransposons and these confer mitotic stability (25). In the MBCs of *T. brucei*, topoisomerase-II activity is also focused at single loci that encompass regions between directional gene clusters that contain transposable elements (21). *T. brucei* Topo-II nuclear isoform sequences have been found to be highly conserved over most of their length with differences in the carboxyl terminal regions (CTRs, i.e., amino acids 1165-1455) which are usually essential for enzyme activity (26). Furthermore, a lot remains to be known about minichromosomes which are composed of 177 base pair repeats and circular DNA of 400 kb, called NR (NlaIII repeat) elements which are common in many strains of *T. brucei* (21, 27, 28).

VSG recombination has been shown to rely on at least two distinct DNA-repair pathways, i.e., RMI1-TOPO3α to suppress recombination and the other which is dependent on RAD51 and RMI1, thus demonstrating their role in antigenic switching (29). Furthermore, the universal mini-circle sequence binding proteins (UMSBPs) conserved at the replication origins of the mitochondrial (kinetoplast) DNA of trypanosomatids have been found to be important in function and integrity of telomere (30). This is of importance because cell division in *T. brucei* is complex due to novel evolutionary and trypanosome-specific molecules acquired over the years, although proteins regulating cytokinesis initiation and completion have not changed (31, 32). Progress has been made on understanding the timing of these events in the cell cycle (33); however, identification of the important proteins involved remains to be established.

### Animal African Trypanosomiasis (Nagana)

This is caused by trypanosome species, the salivaria group, i.e., *Trypanosoma vivax*—subgenus Duttonella, *Trypanosoma congolense*—subgenus Nannomonas and *Trypanosoma brucei specie*—subgenus Trypanozoon whose transmission to the animal host trails is through infected saliva of blood-sucking insects (3).

#### Trypanosoma vivax

*T. vivax* can be found in the wildlife of South America, acting as a reservoir of infection (Table 1). As a result, *T. vivax* infection is considered an emerging illness in South America, although infections have been reported in Mauritius (beyond Africa) due to the proliferative livestock trade (34). It affects mainly bovines, ovines, caprines, *Camelus dromedarius*, and equines and it is genetically different from other animal trypanosomes. For example, *T. vivax* does not proliferate in the vector midgut, rather it prefers the proboscis where it completes its lifecycle. This implies that mechanical minges including *Tabanus* spp. and *Stomoxys* spp. can transmit *T. vivax* (3). Generally, *T. vivax* is maintained in the host vascular system; however, some few exceptions occur where it has been found in the lymphatics, cerebrospinal fluid, and eyes of infected patients (due to adaptions to low oxygen consumption), making chemotherapeutical treatment challenging and development of drug resistance (35).

**Table 1:** Characterization of subspecies and subgroups within *Trypanosoma brucei*.

**Pathogen**	**Location**	**Host**	**Vector**	**Genetic diversity**	**SRA gene**
*T. b. brucei*	Tropical Africa	Both wild and domestic mammals, no humans involved	Tsetse	Extremely high	Absent
*T. b. rhodesiense*	East Africa	Humans, wild and domestic mammals	*Morsitans* group tsetse	Very high	Present
*Trypanosoma brucei gambiense* Group 1	West and Central Africa	Humans, wild and domestic mammals	*Palpalis* group tsetse	High	Absent
*T. b. gambiense* Group 2	Ivory Coast	Humans, wild and domestic mammals	*Palpalis* or *morsitans* group tsetse?	Very high	Absent
**Pathogen**	**Genotype**	**Location**	**Host**	**Vector**	**DNA band**	**KDNA mini-circle size**
*Trypanosoma congolense*	Savannah	Tropical Africa	Wide range of ungulates and other mammals	*Morsitans, palpalis*, and *fusca* groups	369 bp	850 bp
	Forest	West and Central Africa	Pigs, goats, cattle, dogs, other?	*Palpalis* group	~350 bp	850 bp
	Kilifi or Kenya coast	East Africa	Cattle, sheep, goats. Not pigs. Other?	*Morsitans* group	368 bp	700 bp
*Trypanosoma simiae*		Tropical Africa	Suids	Morsitans, palpalis, and fusca groups	521 bp	900 bp
	Tsavo	East Africa	Suids	Morsitans and fusca groups	~540 bp	–
*Trypanosoma godfreyi*		Tropical Africa	Suids	Morsitans and fusca groups	373 bp	800, 750 bp

#### Trypanosoma congolense

These are the smallest trypanosomes and are divided into 3 subgroups, i.e., Savananah, Forest, and Kilifi (36, 37). In cattle, the Savannah subgroup is the most virulent and clinically significant (3) than *T. vivax*. In tropical Africa, *Trypanosoma* (Nannomonas) *congolense* are the most common pathogenic trypanosomes in tsetse flies (38) and it has also been found in horses, sheep, goats, pigs, and dogs. *T. congolense* parasites are limited to the vascular system in their vertebrate hosts, where they use their flagellum to adhere to circulating erythrocytes and endothelial cells, inflicting damage at the adhesion point (3). *T. congolense* is a tiny trypanosome in the mammalian circulation, having a shorter length than *T. brucei* and no visible undulating membrane. Mechanical transmission of *T. congolense* has been demonstrated in laboratory conditions and hence cannot be ruled out as a factor in its spread in Africa (39, 40).

Resistance in *T. congolense* is associated with differences in carbohydrate metabolic pathways as observed in *T. brucei*. *T. congolense* has high comparable oxygen consumption rates as *T. evansi* and *T. brucei* groups although the rate of glucose consumption is lowest in *T. congolense* leading to the production of lactic acid, pyruvate, and carbon dioxide (3). Metabolic inhibitors, i.e., cyanide, malonate, antimycin A, and a combination of cyanide and malonate plus a combination of antimycin A and malonate can inhibit the rate of oxygen consumption by procyclic forms of *T. congolense* using proline as a substrate (41). Proline dehydrogenase, α-ketoglutarate dehydrogenase, succinate dehydrogenase, fumarase, NADP-linked malic enzyme, alanine aminotransferase, and malate dehydrogenase were among the enzymes engaged in proline catabolism with high activity. Alanine and glutamate are the end products of proline metabolism. Aspartate was absent for *T. congolense* unlike in *T.b. brucei* (42). Therefore, glycolysis is important in both *T. congolense* and *T. brucei* for energy metabolism (43). Furthermore, the electron transport chain (ETC) is not required in *T. congolense* followed by substantial resistance to fatty acid synthesis inhibitors (44, 45). These findings raise major challenges for chemotherapy development against drug resistance and host-pathogen interactions. Finally, *T. congolense* lacks an alternative for the *T. brucei TbAT1* gene, which codes for the *P2* nucleoside transporter which is essential for diminazene aceturate absorption (46).

#### T. simiae

*T. simiae* is part of the Nannomonas subgenus as is *T. congolense* and it is the only trypanosome species that is particularly harmful to pigs, causing hyperacute, lethal infection with death occurring within 2 days after the onset of symptoms (47). The parasite can also be found in other domestic animals (48), therefore all livestock on the farm should be treated prophylactically. The pathogenicity of trypanosome infections varies greatly based on a variety of factors, including parasite characteristics (species and virulence), host characteristics (species, breed, age, immunological status, nutritional status, presence of co-infection, and physical condition), vector characteristics (species, density, infection rate, and host preference), epidemiological situation (endemic or epidemic), and the environment (e.g., the availability of food and water and the season) (3).

#### Trypanosoma brucei

There are three morphologically identical subspecies due to host range, diversity, and geographical dispersal other than biological variations, i.e., *T.b. brucei, T. b. gambiense*, and *T. b. rhodesiense*. The fact that *T. b. brucei* and *T. b. rhodesiense* might differ by as little as the expression of a single gene is particularly intriguing. In fact, there is more genetic variety among *T. b. brucei* isolates than there is between *T. b. brucei* and *T. b. rhodesiense* (49). This implies that a majority of *T. b. gambiense* constitute a homogenous group due to the narrow genetic range. In contrast to the normally fast-growing *T. b. brucei/T. b. rhodesiense* phenotype, *T. b. gambiense* group 1 adheres to the conventional understanding of *T. b. gambiense* as a slow-growing parasite in experimental mice (50).

*Trypanosoma brucei* species includes both animal (*T. b. brucei, T. b. evansi, T. b. equiperdum*) and human (*T. b. rhodesiense, T. b. gambiense*) infective subspecies unlike *T. vivax* (at least most strains) or *T. congolense*, which are prevalent in both the vascular system and other organs, and may parasitize the brain in experimental infections (2, 39). The bloodstream form of *T. brucei*, which causes sleeping sickness in people, and the procyclic form, which is found in tsetse flies, have two separate proliferative phases. The species' subspecies *T. b. brucei* predominantly affects cattle and occasionally other animals, and it does not infect humans under normal circumstances. *T. brucei* produces nagana, a wasting illness in cattle, but not in humans. Trypanosomes of the *T. brucei* group (*T. b. brucei, T. b. evansi, T. b. equiperdum*) are morphologically similar (except for the non-proliferative stumpy form in *T. b. brucei*). During chemotherapy, the treatment goal is the trypomastigote infective stage (3).

##### Trypanosoma b. brucei

*T. b. brucei* may be found in several domestic ungulates, though more severe in horses, dogs, and camels. In areas where several trypanosome spp. exist, mixed infections in livestock are widespread, and modern genetic methods make speciation simpler. Many African wild animal species carry at least one species of trypanosome, thus serving as potential reservoirs for highly infectious trypanosomes in humans and livestock (Table 1).

##### T. b. evansi

*T. b. evansi* exhibits features of slender Trypanozoon parasites in fresh blood samples. It is small sized compared to *T. theileri* though it is larger than *T. congolense*. *T. evansi* has previously been understood as a monomorphic thin trypomastigote parasite once viewed on a Giemsa stained thin smear (51). *T. b. brucei* is assumed to be the origin of *T. evansi*, however, it cannot complete its cycle in *Glossina* due to the loss of the maxi-circles of kinetoplastic mitochondrial DNA (51). *T. b. evansi* has acquired a mechanical transmission method which has enabled its expansion and spread rapidly outside of Africa through the export of diseased animals. As a result, it is now the deadly animal trypanosome with the widest geographical range, spanning North-East Africa to most of East Asia and Latin America (51), and it is rapidly spreading. Recently, this infection has been imported in Europe with documented epidemics in Germany, Canary Islands, France, and mainland Spain. *T. b. evansi* may infect both wild and domestic animals (51), however, related studies show that *T. b. evansi* and other trypanosomes, such as *T. lewisi*, a rat pathogen also found in atypical human illnesses, both have a reservoir host in common, i.e., rodents (52). These discoveries have resurrected the importance of rodents as reservoirs of *T. brucei*. Rare incidences of *T. b. evansi* infections in humans have been described and also have the ability to infiltrate host tissues (53) indicating illness being linked to a null genetic mutation in the trypanosome lytic factor blood component Apolipoprotein L1 (APOL1). This generally shields humans against animal trypanosome infections (54), however, no alterations in APOL1 have been detected to elucidate atypical illness (53).

##### T.b. equiperdum

It is widespread throughout Africa and Asia, as well as the Middle East, South America, and Southeast Europe (Table 1). *T. b. equiperdum* has already been exterminated from Western Europe over the last century (55), however, there is still a danger of recurrence, as evidenced by a previous epidemic in Italy (56). The cycle of transmission for *T. b equiperdum* entirely excludes invertebrate vectors. Alternatively, it is passed on through generations of horses and other equids during mating. *T. b equiperdum* unlike other trypanosomes, has vertical or perinatal transmission *via* reproductive organs, resulting in a venereal illness known as dourine. *T. b. equiperdum* is a peculiar protozoan since it mostly invades host tissues and dwells in the blood capillaries of the urogenital tract and very occasionally in the peripheral circulation (57). As a result, parasite diagnosis, isolation, and therapy are all made more complex. Because of this peculiar transmission method and the lack of a reservoir in other species, the disease's management techniques differ from those for other arthropod forms of trypanosomiasis. Furthermore, pharmaceutical therapy is not recommended since it may only relieve clinical symptoms but not completely cure the parasite, thus turning the infected mammal into a possible parasite carrier. While there is no recognized therapy for dourine, studies have shown that melarsomine is effective in treating acute and chronic trypanosomiasis illnesses in horses caused by *T. b. equiperdum* (58).

#### *T. b. evansi* and *T. b. equiperdum*

These are considered as *T. brucei* mutants that have lost some portions of kinetoplast DNA (kDNA) (dyskinetoplastic) or all of it (akinetoplastic). The kDNA is a matrix of circular concatenated mini- and maxi-circles that makes up the mitochondrial genome. In the fly, *T. b. equiperdum* and *T. b. evansi* are unable to complete their life cycle and are trapped in the trypomastigote stage, a stage known to be reliant on glycolysis for ATP synthesis.

Because of a compensatory mutation in the nuclear genome-encoded-component of the ATP synthase, these species of trypanosomes can sustain their mitochondrial function in the absence of the F0–A6 subunit and hence live without the kinetoplast genome (59). As a result, these protozoa are no longer dependent on the tsetse fly for dissemination to their susceptible hosts.

### Human African Trypanosomiasis

Human African trypanosomiasis (HAT), often known as sleeping sickness, is a parasitic illness spread by vectors and is mainly endemic to sub-Saharan Africa. Humans get infected with the parasites through bites infected with tsetse flies infected with pathogenic parasites acquired either from infected human beings or from animals (3).

In humans, two subspecies of *Trypanosoma brucei*, i.e., *T. b. gambiense* and *T. b. rhodesiense*, are morphologically similar but induce unique illness patterns. *T. b. gambiense* and *T. b. rhodesiense* belong to the kingdom Protista, domain Eukarya, phylum Sarcomastigophora, subphylum Zoomastigophora, class Zoomastogophorea. All other animal-like (or non-photosynthetic) flagellates which locomote utilizing whip-like flagella and eat by pinocytosis or phagocytosis are members of this subphylum and class (3).

*T. b. gambiense* is a parasite that causes chronic African trypanosomiasis (better known as “West African sleeping sickness”) which is widespread in over 24 countries in central and west Africa (60). *T. b. rhodesiense* is widespread in 13 countries in eastern and southern Africa, accounting for <5% of all cases of acute African trypanosomiasis (also known as “East African sleeping sickness”) (60). This infection is known as “African sleeping sickness” in humans because it causes lethargy in the affected persons (3). It is only in Uganda where both kinds of the parasite illness may be found in separate parts of the country (61).

According to a review by Giordani et al. (3), *T. b. gambiense* parasites are mainly reserved in the human host; however, the infection has also been found in both domestic and wild animals. Cattle are the principal reservoir for *T. b. rhodesiense* infection. Other domestic animals (dogs, pigs, and sheep) as well as a variety of game species (warthogs, bushbuck, hartebeest, lions, zebras, impala, waterbuck, and hyenas) are also infected (2). The bloodstream form of trypanosomes is taken along with the blood meal when a tsetse fly bites an infected human (or animal). Trypanosomes in the fly travel to the midgut lumen, convert into the procyclic stage which then migrates to the salivary gland after 2 or 3 weeks. It is at this point that they undergo numerous developmental modifications before maturing into adult infectious metacyclic stage that are injected into the skin of a mammalian host while having a blood meal. The metacyclic trypanosomes ultimately become trypomastigotes spreading throughout the body circulatory and lymphatic systems.

#### Pathogenesis of Human African Trypanosomiasis

Trypanosomes use a unique antigenic variation method to evade the immune system. Although the DNA contains up to 1,000 distinct variable surface glycoprotein (VSG) genes, every trypanosome typically encodes single VSG at a moment. The VSG forms a protective layer for the parasite's other invariant outer membrane elements. As antibodies to the VSG are produced, the parasite changes its VSG expression to a different one, still when more antibodies are created against the newly formed VSG, it shifts to another VSG form, and so forth (3). As a result, B cells are massively polyclonally activated, and an elevated IgM level is a characteristic sign of the disease. Immune activities of B-cells and T-cells are similarly suppressed, albeit without clinical implications. Hyperplasia of the lymph nodes and spleen is caused by high IgM levels and the resulting antigen-antibodies complex, along with lymphocytic proliferation. Trypanosomes invade the blood-brain barrier (BBB) and infect the central nervous system (CNS) at a certain stage throughout this protracted process, resulting in chronic lymphocytic meningo-encephalitis (3).

In 2006, 11,382 instances of Gambian trypanosomiasis were documented across the continent, as opposed to 486 cases of Rhodesian trypanosomiasis (62). The DRC continues to have the greatest prevalence of Gambian trypanosomiasis, with 8,023 cases reported in 2006 (up from 26,318 in 1998) (62, 63). In 2006, Angola recorded 1,105 instances (compared to 8,275 cases in 1997), while Sudan reported 809 victims. In 2006, Uganda recorded the largest prevalence of Rhodesian trypanosomiasis at a rate of 245 cases. Conversely, scarce occurrences of Gambian trypanosomiasis have been reported within developed countries mostly among migrants from Central Africa whereas Rhodesian trypanosomiasis has been reported in visitors traveling from East African game parks (64).

### Establishment of *Trypanosoma brucei* Infection

In the 26-megabase genome of this species, there are 9,068 projected genes, including roughly 900 pseudogenes and ~1,700 *T. brucei*-specific genes. According to huge subtelomeric arrays, the parasite employs 806 VSG genes to escape the mammalian host immune responses. Several VSG genes are pseudogenes that may be utilized to construct functional mosaic genes by ectopic recombination (17).

Hall et al. (65) revealed that the long-term *T. brucei* infection, transmission, and parasite success is all influenced by the interaction between host acquired immunity and antigenic polymorphism of the trypanosome's VSG coat. Around 0.1% of the parasite's cycle results in a transition to a new VSG due to fluctuating activation of hundreds of silent VSG genes and pseudogenes, and that distinct antigenic determinants “mosaic” VSG form by segmental gene transformation between donor variable surface glycoprotein genes or pseudogenes. Therefore, mosaic VSG are responsible for antigenic heterogeneity and long-term infection (65).

The VSG and procyclins are glycosylphosphatidylinositol (GPI)-anchored proteins that cover the surface of trypanosome cells. Despite this, the cellular membrane in the host bloodstream is heavily covered with roughly 10 million VSG molecules (parasites' genome comprises around 1,000 VSG genes). GPI anchoring is vital for parasite's endurance and establishment of infection through imitating the humoral immune response of the mammal.

## Therapeutics of Trypanocides

Trypanocidal medications most often used in sub-Saharan Africa include: Isometamidium chloride, ethidium bromide, and diminazene aceturate accounting for 40, 26, and 33%, respectively (1). Isometamidium has equally prophylactic and therapeutic properties, whereas diminazene aceturate exclusively has curative capabilities. Suramin is employed in the management of *T. b. evansi* infections in comparison to other antibiotics (3). Homidium is one of the key medications currently available to regulate AAT according to Sahin et al. (66). The alarming increase of resistant cases to these small numbers of available trypanocides, particularly diminazene aceturate and isometamidium, is concerning because it implies that their future utility may be compromised.

It has been reported that diminazene and isometamidium drugs are the most commonly used medications to cure animal trypanosomes, however, these cannot penetrate the BBB, thus this might be a significant problem in the management of *T. brucei* parasites not within the circulatory system of its mammalian host (3). In addition, Cymelarsan (melarsamine hydrochloride) administered at a dosage of 0.25 and 0.5 mg/kg body weight has been successful in horses with acute and chronic dourine, respectively, due to its high curative effects (58). This revitalizes demand for research promoting the development of a wider spectrum of trypanocides in AAT.

### Human African Trypanosomiasis and Therapy

International interest for the development of novel chemotherapeutical options is currently weak as observed from the limited drug options for the management of HAT (Table 2). This has subsequently led to the classification of trypanosomiasis as a Neglected Zoonotic Disease (NZD) by the U.S. Department of Health & Human Services through the Centers for Disease Control and Prevention (CDC) and the World Health Organization (WHO) (96–98).

**Table 2:** Approved drugs for the treatment of human trypanosomiasis.

**Drug class**	**Drug name**	**Molecular targets**	**Disease form**	**Disease stage**	**Drug form**	**Dosage**	**Major limitations and side effects**	**Year of discovery**	**References**
**Human African trypanosomiasis (HAT)**									
Diamidine	Pentamidine	Binds to parasite DNA, inhibits type II topoisomerase, and disrupts mitochondrial DNA	Effective against *T. b. gambiense* infection; most used drug for early HAT	First stage	Colorless powder	4 mg/kg/day IM or IV (diluted in saline in 2-h infusions) × 7 d (67–69)	Hypoglycemia, hypotension, drug resistance, reasonably tolerable, yet do not permeate through the BBB **(**therefore only used for treating stage 1 HAT), highly polar and these drugs are available in powder form for parenteral use during treatment of the early stage of disease	1940	(4, 70, 71)
Polysulfonated naphthyl amine	Suramin	Non-specifically binds to L-α-glycerophosphate oxidase	*T. b. rhodesiense*	First stage	Powder and ready-to-use solution	IM or intravenous (IV) route Test dose of 4–5 mg/kg (Day 0) slowly IV, then 20 mg/kg IV (max 1 gm/injection) over several hours on Days 1, 3, 7, 14, and 21 For children, 10–20 mg/kg of Suramin is given, and maximum of 1 g at times considered. In situations of renal toxicity, daily dose and the interval between doses should be adjusted accordingly (67, 68)	Toxicity, e.g., nephrotoxicity, allergic reaction, although reasonably tolerable, but unable to cross BBB **(**therefore only used for treating stage 1 of the disease), highly polar, has a short half-life, and is available in ready-to-use solution or powder form for parenteral use during treatment of the early phase of the disease	1920	(72–77)
Melaminophenyl arsenical (MPA)	Melarsoprol	Inhibition of trypanothine reductase	Both gambiense and rhodesiense infections (*T. b. gambiense* and *T. b. rhodesiense* infections); currently recommended as first-line treatment for the rhodesiense form, rarely used in the gambiense form	Second stage	Ready-to-use solution in propylene glycol	IV route 2.2 mg/kg per day (max 180–200 mg/day) IV × 10 d Likely chance of developing encephalopathic reaction to melarsoprol can be avoided by considering pretreatment with corticosteroid drugs (67, 68, 78–80)	Narrow therapeutic index, highly toxic, reactive encephalopathy Can cross the BBB, has a long half-life of 35 hours, therefore is widely used during treatment of late stages of the disease, but the associated encephalopathy and drug resistance limit its use	1949	(71, 72, 81–86)
Ornithine analog	Eflornithine	Inhibits ornithine decarboxylase	This drug is much less toxic than melarsoprol but is only effective against *T. b. gambiense*; it is generally used in combination with nifurtimox (as part of the nifurtimox-eflornithine combination therapy, NECT) but can also be used as monotherapy	Second stage	IV infusion	IV route	Large doses (400 mg/kg), the regimen is complex and cumbersome to apply, has a short half-life, and these drugs are available in ready-to-use solution for parenteral use during treatment of the early stage of disease	Registered in 1990	(81, 82, 84, 87–91)
NECT	Nifurtimox-eflornithine combination therapy	Synergistic effect of individual drugs	Effective replacement of toxic melarsoprol in stage 2 *T. b. gambiense* infection; simplifies the use of eflornithine by reducing the duration of treatment and the number of IV perfusions	Second stage	Nifurtimox tablets and eflornithine IV infusion	Oral route (nifurtimox) and IV route (eflornithine) Nifurtimox 15 mg/kg per day orally in 3 doses for 10 d, and eflornithine 400 mg/kg/day IV in two 2-h infusions (each dose diluted in 250 mL of water for injection) × 7 d For children weighing below 10 kg, eflornithine should be diluted in 50 mL of water for injection while those with body weights of 10–25 kg, this drug should be diluted in 100 mL of water for injection. If water for injection is unavailable, eflornithine can be diluted in 5% dextrose or saline (67–69) Eflornithine might not be effective in immunosuppressed patients because it is trypanostatic and not trypanocidal	Less effective against *T. b. rhodesiense*	Introduced in 2009	
Nitroimidazole	Fexinidazole	The precise mechanism of action of this drug remains unknown. However, it is suggested that bacterial-like nitroreductases encoded by trypanosomes activate fexinidazole and its M1/M2 metabolites through reduction to form reactive intermediates capable of damaging DNA and proteins (metabolic activation by a bacteria-like nitroreductase)	*T. brucei*, i.e., *T. b. gambiense* and *T. b. rhodesiense*	First and second stage; it is indicated as the first line for the first stage and non-severe second stage.	Oral tablets	Oral route	There is decreased efficacy in patients with severe stage 2 HAT), therefore it should be used in case of no other available treatment options	It is a new oral nitroimidazole drug candidate entering clinical trials for the treatment of sleeping sickness, has recently been identified as a promising new drug for HAT; delivered in 2018; is included in 2019 in the WHO essential medicines list and WHO HAT treatment guidelines.	(71, 87, 92–95)

During treatment of trypanosomiasis, the major factors to be considered are: (1) the form of the disease and (2) the phase of the disease (Table 2). The prognosis is good if treatment is initiated in the initial phases of the disease (with early diagnosis), while the second stage requires drugs able to cross the BBB to reach the parasites (4, 67, 99). Currently, there are four approved drugs for treating HAT, i.e., suramin and pentamidine both used during the first (hemolymphatic) stage, and eflornithine and melarsoprol recommended for the second (meningo-encephalic) stage since this involves neuro-migration of the parasites to the CNS. Major challenges associated with these therapies include the high cost, poor oral bioavailability, toxicity, lack of efficacy, and prolonged treatment (87, 92, 100, 101). For example, Eflornithine involves multiple intravenous (IV) infusions for 2 weeks making it an expensive therapy especially for rural health centers, while melarsoprol is very toxic and usually causes reactive encephalopathy (encephalopathic syndrome) which has been associated with deaths in 3–10% of HAT patients (87). This has created a demand for novel therapeutical options which are more effective, safer, and easy to administer. To address challenges associated with monotherapies, approval of Nifurtimox-eflornithine combination therapy (NECT) is currently being promoted as the standard form of treatment. NECT has been associated with low toxicity, and a shortened therapeutical period unlike eflornithine or melarsoprol monotherapy. However, NECT requires 7-d IV infusions which can be a challenge to adhere to especially in resource limited settings (81). In addition, a 2-substituted 5-nitroimidazole (fexinidazole) is currently undergoing extensive preclinical and clinical trials for its use in HAT and preliminary studies have provided favorable results on safety, effectiveness, short-duration of treatment, oral use, and curing both acute and chronic HAT (93). The first all-oral treatment (fexinidazole) is ingested for 10 d for the treatment of both stages of the most common form of infection (*T. b. gambiense*). It is more advantageous than the previous standard therapy (NECT) since it eliminates frequent hospitalization and reduces the number of lumbar punctures (102, 103).

#### Suramin Mode of Action

Suramin sodium is a symmetric polyanionic sulfonated naphthylamine drug. This trypanocide was launched in 1921 as a therapy of surra in camels and for the management of acute phase HAT. It is the oldest trypanocide currently in use (60). It is usually applied to treat HAT caused by *T. b. rhodesiense*, and as such it is still presently accessible. However, it has been superseded by pentamidine in the management of trypanosomiasis caused by *T. b. gambiense*. This drug is also the usual therapy for trypanosomiasis in horses mainly caused by *T. brucei* spp., outperforming diminazene since it is less toxic compared to quinapyramine.

Suramin forms a strong electrostatic bond with human blood proteins and several trypanosome enzymes. The medication was thought to penetrate trypanosomes *via* LDL receptor-mediated absorption and concentrate in the lysosome (104). This notion, on the other hand, appeared to be dubious and the exact mechanism of action has yet to be discovered. Suramin suppresses ATP generation during the glycolytic pathway in *T. brucei* through blockage of glycerol-3-phosphate oxidase and NAD+-dependent glycerol-3-phosphate dehydrogenase (105). In bloodstream *T. brucei*, 28 genes are involved in suramin action, including a surface glycoprotein family (ISG75), a known drug ligand; cathepsin L thought to deliver drug compounds from the ligand within the lysosomal system; a number of deubiquitinating enzymes, and numerous proteins participate in the endocytic pathway (106).

#### Melarsoprol Mode of Action

It is a member of the triazines family and is generated from arsenic. It has several negative toxic effects, including reactive encephalopathy (encephalopathic syndrome), which has a 3 to 10% mortality rate. It is now prescribed as a first-line therapy for rHAT, but it is only occasionally applied for the management of gHAT (60). This is the only currently available therapy for late-stage infections (parasitemia of the CNS) due to rHAT.

Melarsoprol is a prodrug that is converted to the active compounds melarsen oxide after delivery into the mammalian host. It operates by inhibiting the enzyme pyruvate kinase (PK) required during the parasite's aerobic metabolism of glucose. It works by binding irreversibly with PK protein sulfhydryl groups and neutralizing enzymes which interrupts the trypanosome ATP generation processes. Melarsenoxide (Mel Ox) interacts with trypanothione as well as spermidine-glutathione adduct that substitutes glutathione in parasite. Melarsen oxide-trypanothione adduct (Mel T) inhibits trypanothione reductase competitively, thereby extinguishing the parasite. Enough of the melarsoprol metabolite reaches the cerebrospinal fluid (CSF), where trypanosomes pick it up and concentrate it. This medication is very hazardous, with several adverse effects since it is incapable of discriminating the PK for host and that of the parasite (107).

#### Eflornithine Mode of Action

It is prescribed for treatment of the second stage of sleeping sickness associated with *T. b. gambiense*. This trypanocide can be used in combination with nifurtimox. The lower toxicity exhibited by eflornithine to the host has led to its use as a substitute alternative to melarsoprol as the first-line treatment for HAT (108).

Eflornithine is substantially less toxic than melarsoprol and it is only effective against *T. b. gambiense*. Nonetheless, it is most commonly used in conjunction with nifurtimox (NECT), although it may also be administered alone, despite the fact that the treatment is complicated and time-consuming to administer (60). Due to the severe cytotoxicity associated with the use of this dual medication in the management of *T. b. gambiense*, melarsoprol has been restricted to the management of second-stage *T. b. rhodesiense*. It facilitates the administration of eflornithine by lowering therapeutic time and the number of IV perfusions required. Eflornithine works by inhibiting ornithine decarboxylase, an enzyme that catalyzes the formation of amine-based chemicals important in cell division and differentiation. Currently, combination therapies are already being explored to avert the occurrence of eflornithine-resistant trypanosomes, although little has yet been published in this regard.

#### Nifurtimox Mode of Action

Nifurtimox was first used in the treatment of the second stage of HAT as part of the nifurtimox/eflornithine combination therapy (NECT). However, when compared to other similar medications, nifurtimox alone is regarded as a poor trypanocide (109). Nifurtimox can treat both phases of *T. b. gambiense* including 60–90% of melarsoprol-resistant patients. *In vitro* and *in vivo* studies have revealed that nifurtimox-resistant (NfxR) *T. brucei* is cross-resistant to fexinidazole (110). The mechanism of action of nifurtimox has not been fully elucidated, however, it is believed that nifurtimox causes oxidative stress to the parasite (110). Inhibition of parasite dehydrogenase activity is another mode of action of nifurtimox that warrants further research. The Type 1 nitroreductases in the two-electron reduction of nitroheterocycles induces oxidative stress thus causing cellular death in the parasite (109, 111).

#### Fexinidazole Mode of Action

Fexinidazole is a medical breakthrough that is the first all-oral therapy for HAT caused by *T. b. gambiense* in patients aged 6 years and above who weigh at least 20 kg. It initially obtained a satisfactory scientific opinion from the European Medicines Agency in 2018 (60) and it is currently included in the WHO interim recommendations (102). This compound is recommended for first-stage and non-severe second-stage illnesses. It is administered within 30 min following a solid meal and under the supervision of a physician. A clinical investigation for its usage in rHAT is ongoing (60). The drug is also effective against *T. cruzi* (112), although no cases of American trypanosomiasis have been reported in Africa to this day probably due to limited movements and trade between Latin America and Africa.

*In vitro* studies carried out in *T. b. gambiense* have shown that fexinidazole and its two major metabolites, a sulfoxide (M1) and a sulfone (M2) are very crucial in its antitrypanocidal effects. Thus, it has been proposed that trypanosomes encode bacterial-like nitroreductases that reduce fexinidazole and its M1/M2 derivatives to generate reactive metabolites that can damage the trypanosome genome and its proteins (93). This implies that once the drug is absorbed by the parasite, its metabolites are the ones responsible for its therapeutical effects.

#### Pentamidine Mode of Action

Pentamidine is exclusively utilized in the initial stages of *T. b. gambiense* illness, before transiting to the CNS. It is utilized as a backup alternative to suramin. Pentamidine works by disrupting the critical activities in DNA, RNA, phospholipid, and protein production. This drug creates cross-link between two adenines at 4–5 pairs apart in adenine-thymine-rich portions of *Trypanosoma* DNA. It also suppresses type II topoisomerase in the mitochondria of the parasites, culminating in a mitochondrial genome that is fragmented and unreadable (113).

### African Animal Trypanosomiasis and Therapy

African trypanosomiasis in animals has been associated with critical livestock production losses demonstrating the importance to shift policy on disease control to eliminate reservoir host species in endemic communities. The World Health Organization (WHO) has targeted the elimination of HAT “as a public health problem” by 2020 (114), however, this has remained challenging due to the evolving epidemiology pattern of HAT thus questioning the current status quo that the *rhodesiense* form is zoonotic while the *gambiense* form is the main reservoir in disease transmission (115).

The WHO 2020 target was associated with increased infrastructure and fewer cases being reported showing that its elimination was on track, however, the need to generate innovative tools for control of infections and elimination by 2030 cannot be taken for granted (116–118). This is because there has been slow progress in clinical isolates for *gambiense* HAT (114) and for *rhodesiense* HAT (117), however, this situation is geographically specific (for African countries) and not diffuse demonstrating challenges for the attainment of the 2030 target. Infections in domestic livestock [see Kasozi et al. (9)] in small ruminants which are never screened in several developing countries) will continue to act as sources of sporadic infections in humans creating a need to revise the current disease control strategy (Table 3). Furthermore, the narrow chemotherapeutical spectrum available for the control of AAT will continue to undermine WHO HAT targets unless policy is driven to promote innovations in the pharmaceutical industry to control infections.

**Table 3:** Drugs for the treatment of animal trypanosomiasis.

**Drug class**	**Drug**	**Molecular targets**	**Disease form**	**Drug form**	**Route of administration**	**Major limitations and side effects**	**References**
Phenanthridine	Homidium, isometamidium	Inhibits topoisomerase-II during DNA biosynthesis	Prophylaxis and treatment of *T. evansi, T. vivax, T. congolense*, and *T. vivax*; widely used in the treatment of animal trypanosomiasis	Powder for reconstitution	IM route	Highly toxic, drug resistance, highly polar, and these drugs are available in powder form for parenteral use during treatment of the early stage of disease	(119–121)
Aminoquinaldine	Quinapyramine	Trypanostatic, inhibits kinetoplastic DNA biosynthesis, loss of ribosomal function	Effective against *T. congolense, T. vivax, T. brucei*, and *T. evansi*	Powder for reconstitution	IM or IV route	Serious local reactions at site of injection, drug resistance	(122–124)
Diamidine	Diminazene	Inhibition of the kinetoplasmatic DNA biosynthesis	Treatment of *T. evansi*; widely used to treat animal African trypanosomiasis; most used drug for early animal African trypanosomiasis (AAT)	Powder for reconstitution	IM or IV route	Highly polar, poor permeation, poor brain permeation due to its cationic polar nature, although it is well tolerated, it requires repeated administration, leading to poor patient compliance	(125–128)
Melaminophenyl arsenical	Melarsomine	Inhibition of trypanothine reductase	*T. evansi* infection	Powder for reconstitution	Administered by IV or IM route	Rapidly metabolized in the plasma	(129–132)

#### Phenanthridine and Mode of Action

Homidium bromide, also known as ethidium bromide, was launched as an advancement over earlier phenanthridine-based trypanocidal agents and it is also accessible as a chloride salt (133). Cross-resistances are attributed to the phenanthridine core. Homidium may perhaps be prescribed as a sanative pair with diminazene aceturate, but never with isometamidium (134). Treatment with this antitypanocide causes dyskinetoplasty in the same manner as several other phenanthridines and diamidines do (135) and alteration in gene activity has been believed to be a major attribute to its trypanocidal effects. Moreover, by twisting and altering the double helix structure, homidium inhibits both kinetoplast and nuclear DNA replication in *T. brucei* (136). At low dosages (0.02 g/ml), homidium was shown to eliminate dyskinetoplastic trypanosomes primarily through the breakdown of the kinetoplast network. However, at larger doses, homidium disrupts the parasite's gene, which explains its capability to kill dyskinetoplastic trypanosomes.

Isometamidium chloride hydrochloride contains therapeutic as well as preventative qualities used in the management of African trypanosomiasis. It is a blended phenanthridine with amphiphilic and cationic characteristics that are made by combining homidium with the diazotized *p*-aminobenzamide moiety of diminazene and then modifying it with the amidine group in the meta position. The phenanthridine isometamidium is primarily employed in the initial stages of AAT. The isometamidium chloride formulations mainly include a mixture of four phenanthridine compounds, namely; isometamidium chloride hydrochloride [8-(3-mamidinophenyl-2-triazeno)-3-amino-5-ethyl-6 phenylphenanthridinium chloride hydrochloride], the positional red isomer [3-(3-m-amidinophenyl-2- triazeno)-8-amino-5-ethyl-6-phenylphenanthridinium chloride hydrochloride], the blue isomer [7-(mamidinophenyldiazo)-3,8-diamino-5-ethyl-6 phenylphenanthridinium chloride hydrochloride], and the disubstituted compound [3,8-di(3-m amidinophenyltriazeno)-5-ethyl-6-phenylphenanthridinium chloride dihydrochloride]. Regrettably, this trypanocide is unsuccessful in the treatment of trypanosomiasis illnesses caused by *T. b. evansi*, hence it is less widely utilized beyond sub-Saharan Africa (60, 137). Isometamidium chloride works by forming an unconventional “sideways” geometry bond to kDNA of the trypanosome (138).

#### Aminoquinaldine and Mode of Action

Surfen C is one of the early trypanocides from which quinapyramine sulfate was developed (139). The mechanism of action of quinapyramine is uncertain, however, two hypotheses have justified its interference with DNA synthesis and suppression of cytoplasmic ribosomes (hence, inhibit protein synthesis). However, like with phenanthridines and bis-benzamidines, its dicationic/aromatic properties imply mitochondrial drug accumulation as its mode of action (3).

#### Diamidine and Mode of Action

Studies have shown significantly less efficiency of diminazene aceturate against the *T. congolense* group as compared to the *T. b*. group. This could be attributable to the absorption route used by latter parasites *via* the P2/TbAT1 transporter that permits for faster and concentrated absorption. Diminazene aceturate is promptly metabolized and eliminated, thus it is more ideal as a curative drug than prophylactic use (3). Diminazene aceturate binds to the minor groove of the DNA AT-rich locations of the parasite. kDNA is the recognized target of this drug where it binds, ultimately inducing inhibition of replication and kDNA loss. This may be worsened by the effects caused by suppression on mitochondrial type II topoisomerase (3), leading to a disruption of mitochondrial membrane transport protein activity. Furthermore, *in vitro* studies reported by Gould and Schnaufer (140) highlighted that trypanome dyskinetoplastic lines showed substantial resistance to diamidines, i.e., diminazene aceturate and phenanthridines.

Diminazene aceturate has been proposed to modify the reactions of the host's immunity by reducing pro-inflammatory cytokines and overwhelming immunological stimulation, which might impact the drug's *in vivo* properties (141). Diminazene aceturate can only pass through the cell membranes through specific transporters due to its charged composition, and this implies that: (a) The medicine is ineffective against infections that advance to the CNS because it cannot pass through the BBB; (b) trypanosomes express carrier proteins that particularly express genes to cause resistance; and (c) a loss of these specific transports by the parasite through VSG activity inevitably leads to induced resistance.

#### Melaminophenyl Arsenical Mode of Action

Melarsomine dihydrochloride is a melamino-phenylarsine that is produced by combining melarsen oxide with two cysteamine equivalents. Compared to melarsoprol, the chemical exhibits a higher water solubility (3). The drug (or, more precisely, its metabolite melarsen oxide) penetrates the cells of *T. brucei via* the same P2/ TbAT1 adenosine nucleoside transporter and TbAQP2 transporters that also deliver similar trypanocides; melaminophenyl arsenicals and diamidine (142, 143) highlighted that the majority of the cytotoxic activity of arsenicals is presumably due to selective absorption.

## Trypanocidal Resistance

Close to 35 million doses of trypanocides are delivered in sub-Saharan Africa each year and are only adequately able to care for about one-third of the animals at risk (1). Remarkably, farmers in Africa have consistent access to a majority of the trypanocides which has culminated in widespread overuse and under-dosage of drugs without the guidance of veterinarians, resulting in the evolution of trypanocidal drug resistance. This situation has been precipitated by the lax legislation which promotes liberalization of the drug industry; however, this has led to increased drug abuse, wastage, and development of drug resistance.

At the moment, trypanocidal resistance has been registered in 21 African nations (1) including Ethiopia (6, 58). A study done between 1996 and 2003 in the Eastern Province of Zambia documented a five-fold significant increase of diminazene aceturate resistance to *T. congolense* over the 7-year study period (144). The usage of similar trypanocides over time has put selection pressure on drug-targeted trypanosome genes, resulting in genome mutations which promote trypanocide resistance (Table 4).

**Table 4:** Anti-trypanosomal drugs, transporters, and drug resistance.

**Drug name**	***Trypanosoma* transporter**	**Mechanism of trypanocide resistance**	**Type of drug resistance**	**References**
Nifurtimox	*P2* aminopurine transporter which is encoded by *TbAT1* gene	Loss of the *P2* aminopurine transporter which is encoded by the gene *TbAT1* Changes in *TbAT1* through, deletions, chimeric rearrangement, and mutations	Resistance to nifurtimox	(145–148)
Suramin	Endocytic pathway invariant surface glycoprotein *ISG75*	Loss of function of the suramin receptor (ISG75) Also involves loss of function for lysosomal proteases (Cathepsin L), endosomal proteins, and a lysosome-based major facilitator superfamily (MFST)	Resistance to suramin	(145, 146, 148, 149)
Melarsoprol	*P2*-purine transporter	Resistance mechanisms for the MPA melarsoprol are similar to that of the diamidines due to similar motifs in the benzamidine moieties and melamine-phenyl group; therefore, “impairment in permeability to, or affinity of, the melamine grouping in the melarsen-resistant strain also prevents the uptake of the “amidine-type drugs”	Melarsoprol/pentamidine cross-resistance (*MPXR*)	(145, 146, 148, 150, 151)
Eflornithine	*AAT6* (amino acid transporter 6) encoded by the gene *TbAAT6*	A single point mutation, e.g., deletions of the gene *TbAAT6* are associated with significant drug resistance	Resistance to eflornithine	(145, 152–154)
Diamidine: Pentamidine (most used drug for early HAT)	Aquaglyceroporin 2 (*TbAQP2*) Additional pentamidine/arsenical transporters: high-affinity pentamidine transporter (*HAPT1*) and low-affinity pentamidine transporter (*LAPT1*), encoded by *TbAQP2* and *TbAQP3*	Loss of the *P2* aminopurine transporter encoded by the *TbAT1* gene Deletions, mutations, and chimeric rearrangements with the adjacent *TbAQP3* gene causes changes in *TbAQP2*	*MPXR* Selective resistance to pentamidine and high-level *MPXR*	(145, 146, 148, 151)
Diamidine: Diminazene (most used drug for early AAT)	*TbAT1/P2*	Loss of the P2 aminopurine transporter encoded by the gene *TbAT1*, also chimeric rearrangements, mutations, deletions with *TbAQP3* gene that is adjacent causes changes in *TbAQP2*	Resistance to the veterinary diamidine diminazene, melarsoprol/pentamidine cross-resistance (*MPXR*)	(145, 146, 148, 150)

Unfortunately, there has been a dearth of new trypanocides for decades, culminating in a condition where the limited number of drugs known to be accessible have diminishing efficacy as drug-resistant genes emerge (3). The drugs presently existing in communities have narrow therapeutic indices, yet a majority also cause local pain at the site of injection (especially with isometamidium). Remarkably, irrational usage of the trypanocides in the past has led to the development of drug resistant trypanosome species, and commencement of cross-resistance among almost all existing trypanocides that are chemically related, thus exacerbating the situation. Resistance has also been attributed to having therapeutic usage of most of the trypanocides rather than prophylactic application.

The choice between curative and prophylactic medications is influenced by a number of factors which include but is not limited to: the risk and exposure to the infection, accessibility of the drug, and logistics of delivery (155). Multiple-dose delivery regimens are generally not feasible in impoverished nations, and animal care facilities are often quite restricted. In management of trypanosomiasis in livestock, trypanocides prescribed as single dose required to cure and prevent the infection are usually favored. Unlike with HAT, where NECT is currently the standard first-line treatment for second-stage illness (81, 156), there is no other medication combination utilized in the treatment of AAT.

Henceforth, where possible, interchanging the use of available trypanocides with limited threat of cross-resistance, such as diminazene aceturate and isometamidium (known as a “sanative pair”), has been proposed, however, information on the therapeutics remains scarce to this date. This has subsequently led to medication errors reported in both West and East Africa, against *T. congolense* and *T. vivax* infections with isometamidium and diminazene. Moreover, cross-resistance profiles on diminazene aceturate and isometamidium had been thought to be an uncommon occurrence (3). These numerous resistant strains have been the consequence of distinct selection pressures created in the communities, justifying a need to revise policy to promote biomedical research to identify novel pathways which could widen the therapeutical options for the twenty-first century.

To preserve the efficacy of presently utilized drugs, it is critical to justify drug dose regimens based on whether the trypanosome species found in a specific region are phenotypically susceptible to trypanocide. Amid the continuing effort to develop new therapeutic and prophylactic trypanocidal drugs through Global Alliance for Livestock Veterinary Medicines (GALVmed), a public–private partnership with financial support from the Bill and Melinda Gates Foundation and the UK Department for International Development (http://www.galvmed.org/en/), no novel authorized drug has been developed for the last 50 years, necessitating the urgency to focus on innovative drug discoveries. Notwithstanding this need, a majority of the pharmaceutical industries have been discouraged to further invest drug discoveries due to the elevated expenses required in medicine development and the poor projected return on chemotherapy sales in developing countries (3).

Trypanosome survival within their mammalian and insect hosts has been associated with evolutionary changes involving restructuring of the VSG. Transitional changes during the trypanosome lifecycle implies that innate and acquired immune responses must be eluded through antigenic variation of the VSG which are present on the surface of the parasites. Currently, chemotherapy is the only available option against trypanosomes as efforts to develop a vaccine have been thwarted by the international community, however, the development of trypanocidal resistance has become a realistic threat especially for persons living in endemic regions (103, 145, 157, 158).

*Trypanosoma* transport proteins [on the surface of the parasite (see Table 4)] are responsible for pathogen survival; and trypanocidal resistance has been associated with changes in the pathogen transportome leading to dysfunction and a loss in therapeutical potential once drugs can no longer be absorbed by the parasite (152–154, 159). This justifies the need to promote further research for the discovery of novel chemotherapeutical options for the control of trypanosomiasis.

### Diminazene Aceturate Mode of Resistance

Drug resistance to diminazene aceturate is prevalent mostly in *T. congolense* infections, and it has been related to a mutation that alters the activity of a P2-type purine-transporter involved in drug absorption (1). Diminazene resistance was found in *T. brucei* subspecies *brucei, evansi*, and *equiperdum* when P2/TbAT1 expression was lost. Moreover, an additional gene called TeDR40 has been linked to resistance in *T. b. evansi* (160). TbAQP2 does not appear to be functional in *T. congolense*, however, TcoAT1, a potential P2/ TbAT1-type carrier protein with specific allele linked to diminazene aceturate resistance was discovered in *T. congolense* (161). Other chemically incomparable substances such as suramin and quinapyramine showed no cross-resistance. However, no new trypanocides have been developed despite the rising cases of trypanocide resistance.

### Homidium Salts Mode of Resistance

Studies done on *T. brucei* show that homidium is assimilated into the kinetoplast and nucleus of trypanosomes. However, it is still unknown why trypanosomes establishes resistance to homidium, even though it is thought to be comparable with that of isometamidium, a compound related to it (136).

### Isometamidium Chloride Mode of Resistance

Isometamidium can be used in a sanative combination with diminazene, with the two medications being given in a certain order to reduce the chance of emerging resistance. Several African countries have demonstrated rising resistance to isometamidium especially in *T. congolense*, followed by *T. brucei* and *T. vivax* species and occasionally revealing cross-resistance with diminazene aceturate (162).

This is contrary to previous findings (1990s) when isometamidium was the most efficacious agent against AAT and no resistance was ever reported (163). After 15 years, extreme resistance to isometamidum chlodride in *T. b. brucei* without a functioning kinetoplast, as well as naturally occurring dyskinetoplastic *T. b. evansi*, has become rampant.

A study by Dean et al. (59) showed that the loss of the kinetoplast in trypanosome cells does not affect the mitochondrial membrane potential since a compensatory mutation in the F1F0-ATP synthase, a mutation in this ATP synthase subunit is adequate to generate a significant amount of resistance to isometamidium and homidium. The resistance mechanism in *T. congolense* has been associated with reductions in mitochondrial function. As a result, drug build-up in the mitochondrion is minimized, resulting in decreased absorption through the plasma membrane, due to the quick equilibration of drug quantities between the intracellular and extracellular composition of the cell as soon as the mitochondrial sink is removed. Furthermore, active expulsion of the drug *via* plasma membrane transporters is another mechanism of resistance (164), however, few studies have explored these mechanisms further.

### Quinapyramine Sulfate Mode of Resistance

Due to widespread resistance, the drug was discontinued from use in livestock until 1976 (165). It was then restored to use from around 1984 up to date in the management of *T. b. evansi* infections in horses and camels. The quinapyramine-resistant *T. congolense* have cross-resistance to isometamidium, homidium, and diminazene, and thus it is not indicated for usage in livestock (134). Even though the specific mechanism of quinapyramine resistance is uncertain, it is probable that most of these drugs have a mitochondrial target, and that any slight variation in the potential of the parasite's mitochondrial membrane, or when the inner mitochondrial membrane loses its organic cation carrier, multi-drug resistance might occur.

### Suramin Sodium Mode of Resistance

Although suramin is effective in the treatment of trypanosomiasis caused by *T. simiae* in pigs, it is unsuccessful in the management of *T. vivax* and *T. congolense* infections, owing to the metabolic physiological variations that differentiate *T. brucei* group species from other groups. By inhibiting suramin uptake, or its typical transit *via* the endocytic route after attaching to a particular receptor, it seems enough to make parasites resistant to the medication, although it is still uncertain exactly how suramin kills trypanosomes once aggregated intracellularly.

### Melarsomine Dihydrochloride Mode of Resistance

Reduced P2/TbAT1 activity is a recognized cause of cross-resistance in melarsomine dihydrochloride that penetrates the trypanosomes through this route. *T. brucei* strains resistant to melarsomine have a generally reduced susceptibility to diamidines and other arsenical medicines like melarsoprol, however, not to suramin (166, 167). *T. congolense* and *T. vivax* lack legitimate orthologs of TbAT1 and TbAQP2, which might suggest why the drug is less effective against such parasites (see Section Diminazene Aceturate Mode of Resistance).

### Melarsoprol Mode of Resistance

The adenine-adenosine transporter, P2, is the source of resistance arising from point mutations within this transporter. The inactivation of this transporter is connected to trypanocide resistance (107). Furthermore, Koning (168) reported that over-expression of the gene encoding for TbMRPA, P-glycoprotein efflux pumps promote resistance to melamine-based arsenicals by causing the trypanothione adduct to develop within the cell to be excreted. Resistance is due to loss or point mutations associated with the P2 transporter (107). Furthermore, Barrett et al. (109) reported that for high levels of melaminophenyl arsenical resistance, both P2 and HAPT1 carrier proteins must be lost and of which the two resistance mechanisms were found to act independently but were rigorously complementary in studies.

### Eflornithine Mode of Resistance

Resistance to eflornithine is caused by the deletion or mutation of the TbAAT6 gene in many trypanosomes (108, 146). This gene is conserved throughout the *Trypanosoma* genome and encodes for the transmembrane amino acid transporter that delivers eflornithine into the cell, thus loss of the transporter protein results into eflornithine resistance. Resistance to eflornithine has potentiated the usage of melarsoprol which has later been associated with a 5% mortality rate among HAT recipients due to its toxic effects (109, 169).

### Nifurtimox Mode of Resistance

The loss of a single copy of the genes encoding for nitroreductases enzymes (NTR) attributes to nifurtimox resistance (110, 111). Few studies have been conducted to explore resistance mechanism any further to date.

### Pentamidine Mode of Resistance

Pentamidine enters the mitochondria *via* the carrier proteins *P2* and *HAPT1* transporter proteins, thus loss of any of these carrier proteins results in resistance to pentamidine (109, 113).

## New Antitrypanosomal Compounds in the Pipeline

The lack of a vaccine against HAT subspecies and antigenic variation of *T. brucei* makes treatment very challenging and expensive (170, 171). Limitations of monotherapies such as severe side effects and development of resistance imply that emphasis in chemotherapy is on combination therapy as observed with NECT which has continued to enjoy WHO approval (84, 170) (Table 5). Currently, new proposed candidates include nitroimidazole analog fexinidazole, i.e., 5-nitroimidazole, and its principal metabolites (fexinidazole sulfoxide and fexinidazole sulfone) have been characterized and shown to have potential for effective oral treatment against both stages of the *T. b. gambiense* and *T. b. rhodesiense* infections (93, 170, 172). In addition 1-aryl-4-nitro-1H-imidazoles (stage II HAT) have a genetic safety in mammalian cells (173–176), although information on clinical trials from endemic areas to revalidate this is scarce. Furthermore, another new drug on the horizon is Oxaborole SYX-7158, which is a benzoxaborole (stage II HAT) and has cleared the parasites in mice (170, 177).

**Table 5:** Current alternative therapeutical options for management of trypanocidal resistance.

**Trypanocidal agent**	**Trade names**	**Structure**	**Route of administration**	**Dosage (mgkg** ^**−1**^ **)** ^**b**^	**Targeted trypanosome species**	**Adverse/side effects**	**Management of relapses**
Diminazene aceturate	Berenil, Ganaseg Trypan Veriben Azidin Pirocide,	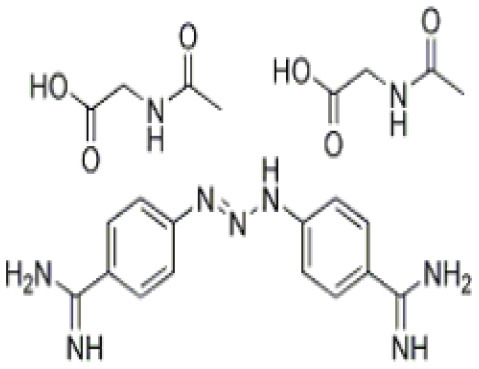	IM	3.5, 8 for resistant trypanosomes, 5-10 for *T.b.evans*.	*T.congolense, T.vivax* Less effective on *T.b.brucei* and *T. evans*	Has toxic effects in camels, horses, donkeys, and dogs, camels.	Isometamidium chloride
Isometamidium chloride	Veridium, Trypamidium, Securidium Samorin,	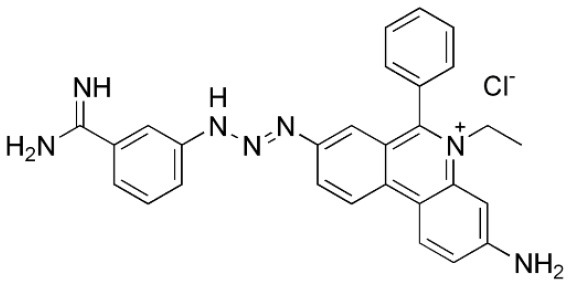	IM	0.25-1	*T. congolense, T. vivax* less effective on *T. b. brucei and T. b. evansi*	Avoid subcutaneous administration. Highly painful, inflammation at site of injection in cattle, and highly cytotoxic at doses above 2mgkg^_1.^	DA
Homidium bromide	Ethidium, Novidium	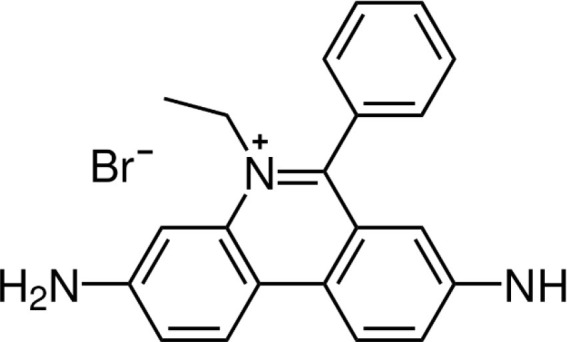	IM, IV	1	*T. vivax T.congolense. low efficacy* on *T. b. bucei* infection *in* livestocks	Administation via IM cause toxic effcets to horses. It is highly carcinogenic	DA and Isometamidium chloride
Quinapyramine sulphate	Antrycide, Triquin-S Trypacide, tribexin, Noroquin, and quintricide,	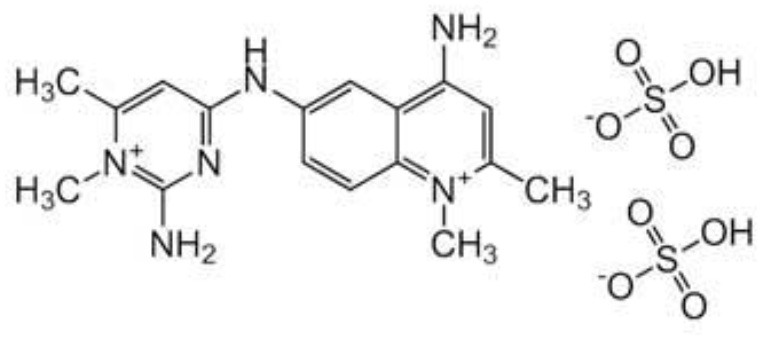	SC	3-5 and 20-40 for *T.simiae*.	*T.b. evansi* *T.vivax* *T. congolense* *T. brucei* *T. b. equiperdum* *T. simiae/* camels	Highly toxic at doses. High chances of resistances	Isometamidium. Suramin sodium
Melarsomine dihydrochloride	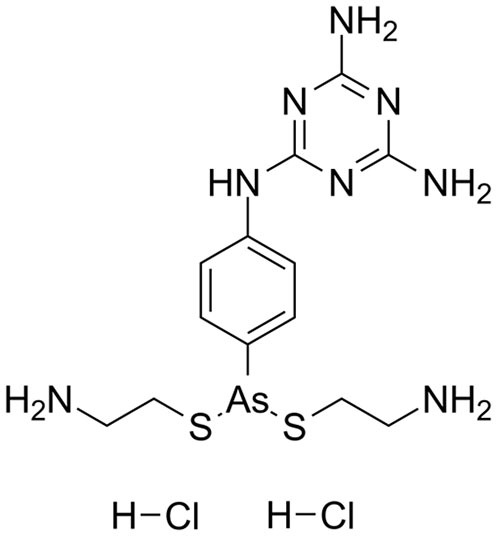	IM	0·25-0.5	*T.b.evansi* in camels	Has self-limiting side effects and immediate	Isometamidium.
Melarsoprol	Mel B, Melarsen Oxide-BAL, Arsobal	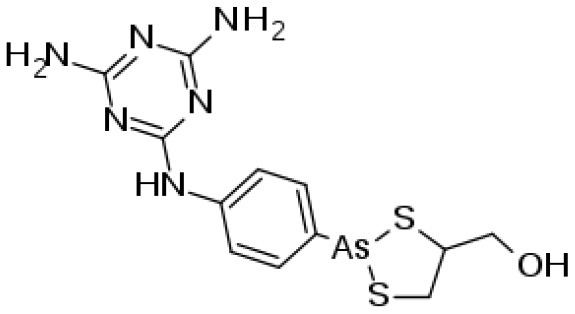	IV, given slowly	2-3.6, given for three days After 1 week: 3.6 for 3 days Repeat again after 10-21 days: 3.6 mg/kg/day (69)	*Trypanosoma brucei rhodesiense*	Administered only in severe cases. Brain dysfunction, encephalopathy, convulsions, loss of consciousness, bloody stools, nausea, fever, vomiting, rashes, numbness, rashes, renal and liver disorders are all common adverse effects. It is not suggested for usage in pregnant women	eflornithine
**Eflornithine**	Vaniqas	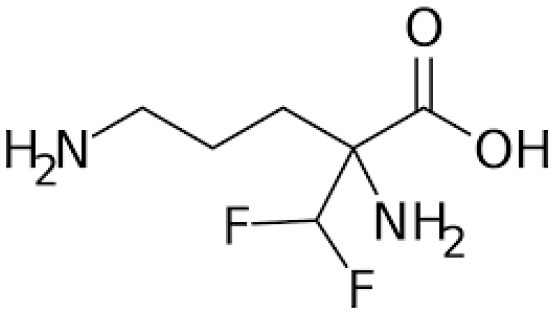	IV, administered slowly/ short tern infusions	100, given at 6 h interval for 14 days - 150, in (children)	T. b. gambiense	Bone marrow suppression resulting into anemia, leucopenia and thrombocytopenia, cancer, and alopecia, hypoacusis Best for patients above 12 years of age	NECT
**Pentamidine** (113) Other names: pentamidine diisethionate, pentamidine dimesilate	For oral inhalation and for nebulizer use: •NebuPent Nebulizer (APP Pharmaceuticals LLC, United States) For IV and IM •United States and Canada: ° Pentacarinat 300 ° Pentam 300 ° Pentamidine isethionate 300 mg	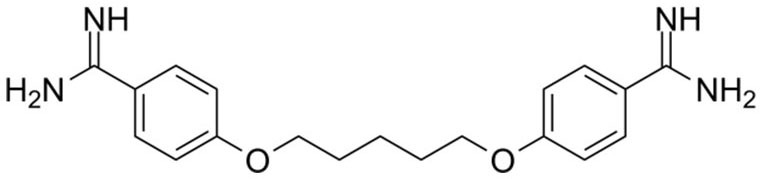	IV, IM, inhalation	4 given IV/IM q d for 14–21 d	For early stage of *T. b. gambiense*	For the injectable formula: low blood sugar, irritation at the sit of injection, vomiting, nausea, low blood pressure, and kidney problems occur. For inhaled formula; patients experience nausea, wheezing, and cough, nausea Others may include; chest pain and skin rash	Eflornithine or melarsoprol
Suramin sulphate	Naganol, Bayer 205, Germanin	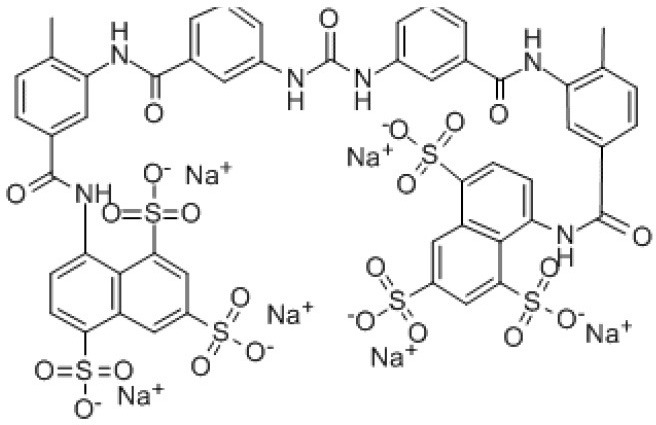	IV, Given as 3 doses per week in horses	A dose of 10mg Taken every 5 days to total of 12 injections for *T. b. gambiense* Adult: 100-200 mg (test dose) (IV), then 1 g IV on days 1, 3, 7, 14, 21 Pediatric: 20 mg/kg- 1 g/dose IV on days 1, 3, 7, 14, 21; not to exceed	*T.b. brucei, T.b. evansi, T.b. equiperdum*	IM causes necrosis at the site. Cause toxicity in horses. Others include; Vomiting, Hives, Numbness and tingling, Nerve pain in extremities, Kidney damage, Blood disorders, pancytopenia, Shock, Optic atrophy	Quinapyramine sulphate
***Nifurtimox***	**Lampit by Bayer**	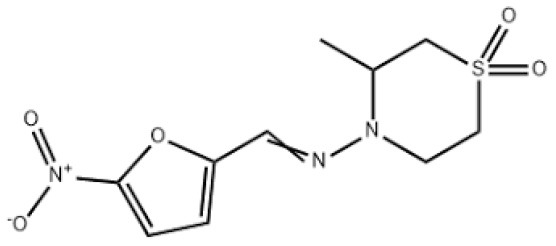	oral	recommended doses are 5 mg/kg per os three times/day for adults and 7 mg/kg three times/day for children for 14–21 days. 30 mg/kg/d for 30 d	***Trypanosoma*** brucei gambiense	Diarrhea, convulsions, vomiting epilepticus and Reversible cerebellar syndrome (ataxia, nystagmus, tremors, and vertigo) was seen with high dose (30 mg/kg/day for 30 days).	NECT

The Drugs for Neglected Diseases initiative (DNDi) has developed an efficacious mixture of two older drugs nifurtimox and eflornithine (81), as well as nitroimidazole fexinidazole (currently left with one clinical trial phase as an oral therapy for second phase HAT).

According to Steinmann et al. (178), a distinct benzoxaborole molecule similar to that used in humans is being developed and progressed for the treatment and prevention of AAT. Several similar agents are being added to the GALVmed portfolio as researchers look for therapies that meet the criteria outlined in a target product profile (TPP), which is applied to determine whatever attributes should trypanocidal agents have to provide a beneficial impact in AAT. The WHO's road map for NZD aimed to eliminate human African trypanosomiasis by 2020 and to cease transmission by 2030 (60).

## Conclusion

African trypanosomiasis is a disease as old as colonial history in most African countries; however, political will both locally and internationally to control and eradicate the disease remains weak. In the previous two decades, great strides were made by global health partners, however, limited progressive research to identify and develop novel therapies has led to the development of trypanocide resistance against the limited chemotherapeutical options in the African market. The WHO has revived interest in the disease, and this has promoted the current ongoing clinical trials. However, reciprocal efforts from the Organization for Animal Health to engage global health partners continues to move at a snail's pace. The development of trypanocide resistance has been explored on the limited therapeutical options; however, a lot of opportunities arise for industry to develop drugs that could help control trypanosomiasis in livestock which are the reservoirs of HAT.

## Author Contributions

KK and SW conceptualized the study. KK, SW, and EM conducted the study design. KK collected the data. KK, EM, IN, and SW conducted data analysis. All authors reviewed, approved publication of the manuscript, and remain in agreement on all aspects of the work. All authors contributed to the article and approved the submitted version.

## Funding

This research was supported by the National Institute for Health Research (NIHR) Global Health Research Programme (16/136/33) using UK aid from the UK government. This work was also supported by Zhejiang University Education Foundation Emergency Research Fund, Global Challenges Research Fund, and the University of Edinburgh (SW and KK). The work was also funded by the Commonwealth Scholarship Commission (grant ID number: UGCS-2021-447) in the UK (KK).

## Author Disclaimer

The views expressed in this publication are those of the authors and not necessarily those of the NIHR or the Department of Health and Social Care.

## Conflict of Interest

The authors declare that the research was conducted in the absence of any commercial or financial relationships that could be construed as a potential conflict of interest.

## Publisher's Note

All claims expressed in this article are solely those of the authors and do not necessarily represent those of their affiliated organizations, or those of the publisher, the editors and the reviewers. Any product that may be evaluated in this article, or claim that may be made by its manufacturer, is not guaranteed or endorsed by the publisher.
